# Franklin's Improved Cup for Taking Impressions of the Lower Jaw

**Published:** 1856-01

**Authors:** 


*Franklin's Improved Cup for taking Impressions of the Lower Jaw.*—The manufacturer, Mr. Franklin, dentist of Newark, N. J., presented to the senior editor, through Dr. Colburn of the same place, an improved cup for taking impressions of the inferior maxillary, in plaster of paris, which he has recently gotten up, and it is more admirably adapted to the purpose than anything of the kind we have ever seen. It is constructed with a concavity or cup on the upper as well as the lower side, with an opening between the two of a quarter of an inch in width, extending nearly from one extremity to the other, except a piece of the mettle with which it is composed, half an inch wide, passes across it in the centre to strengthen the two sides of the cup. The one presented to the senior editor, is made of tin, plated with silver, and is altogether a very pretty affair.

In taking an impression with this cup, the under part is first filled with plaster, mixed in water until of a sufficient consistence to prevent it from running from the instrument; this done, the upper cup is filled immediately. The cup is now at once placed in the mouth and pressed gently down upon the alveolar ridge, until the latter becomes imbedded sufficiently in the plaster; that in the upper side, in the mean time, is pressed with the finger of the operator through the opening in the central part of the instrument, for the purpose of ensuring a perfectly accurate impression. The whole is kept in place, until the plaster has set, or congealed, sufficiently to enable the manipulator, to remove it without breaking—when the impression may be oiled and filled in the usual manner.

After the plaster put in the impression has hardened sufficiently, that in the upper part of the cup may be cut away, and the instrument removed. The plaster is now warmed until it reaches a temperature of about one hundred degrees, when it may be broken away from the model, which will be found to correspond exactly in shape with the alveolar border.

The cup here described, may, we believe, be obtained from Mr. Francis Arnold, instrument manufacturer, Baltimore.

